# Medical crowdfunding in a healthcare system with universal coverage: an exploratory study

**DOI:** 10.1186/s12889-020-09693-3

**Published:** 2020-11-09

**Authors:** Ágnes Lublóy

**Affiliations:** 1 Department of Finance and Accounting, Stockholm School of Economics in Riga, Strēlnieku iela 4a, Rīga, LV-1010 Latvia; 2 Department of Finance, Corvinus University of Budapest, Fővám tér 8, Budapest, 1093 Hungary

**Keywords:** Medical crowdfunding, Universal health coverage, Unmet health care need

## Abstract

**Background:**

In recent years, crowdfunding for medical expenses has gained popularity, especially in countries without universal health coverage. Nevertheless, universal coverage does not imply covering all medical costs for everyone. In countries with universal coverage unmet health care needs typically emerge due to financial reasons: the inability to pay the patient co-payments, and additional and experimental therapies not financed by the health insurance fund. This study aims at mapping unmet health care needs manifested in medical crowdfunding campaigns in a country with universal health coverage.

**Methods:**

In this exploratory study we assess unmet health care needs in Germany by investigating 380 medical crowdfunding campaigns launched on Leetchi.com. We combine manual data extraction with text mining tools to identify the most common conditions, diseases and disorders which prompted individuals to launch medical crowdfunding campaigns in Germany. We also assess the type and size of health-related expenses that individuals aim to finance from donations.

**Results:**

We find that several conditions frequently listed in crowdfunding campaigns overlap with the most disabling conditions: cancer, mental disorders, musculoskeletal disorders, and neurological disorders. Nevertheless, there is no strong association between the disease burden and the condition which prompted individuals to ask for donations. Although oral health, lipoedema, and genetic disorders and rare diseases are not listed among leading causes of disability worldwide, these conditions frequently prompted individuals to turn to crowdfunding. Unmet needs are the highest for various therapies not financed by the health insurance fund; additional, complementary, and animal-assisted therapies are high on the wish list. Numerous people sought funds to cover the cost of scientifically poorly supported or unsupported therapies. In line with the social drift hypothesis, disability and bad health status being associated with poor socioeconomic status, affected individuals frequently collected donations for their living expenses.

**Conclusions:**

In universal healthcare systems, medical crowdfunding is a viable option to finance alternative, complementary, experimental and scientifically poorly supported therapies not financed by the health insurance fund. Further analysis of the most common diseases and disorders listed in crowdfunding campaigns might provide guidance for national health insurance funds in extending their list of funded medical interventions. The fact of numerous individuals launching crowdfunding campaigns with the same diseases and disorders signals high unmet needs for available but not yet financed treatment. One prominent example of such treatment is liposuction for patients suffering from lipoedema; these treatments were frequently listed in crowdfunding campaigns and might soon be available for patients at the expense of statutory health insurance in Germany.

## Background

Crowdfunding is the practice of funding a project or enterprise by collecting small amounts of money from numerous people, typically via online platforms. In the past two decades the market of crowdfunding has been growing quickly; crowdfunding has become a new way to finance, for example, start-up companies, projects in the visual arts and music, technological innovation, scientific research, and community projects.

In the last decade, crowdfunding for medical expenses has gained popularity as well, especially in the United States. Bassani et al. [1] report that 76 medical crowdfunding platforms operating worldwide had raised over $132 million as of October 2017; and that the number of health-related crowdfunding campaigns reached 13,633. In the United States, medical crowdfunding is considered to be a symptom of an inadequate healthcare system; in 2007, 62% of individual bankruptcy filings were related to medical costs due to injury and severe illness [2]. Crowdfunding not only provides relief for a large number of sick people but also helps them to avoid medical bankruptcy [3, 4]. Nevertheless, crowdfunding is a typical tool for obtaining one-off financing; and one-off funding is inadequate to finance chronic diseases and other life-long health problems.

In Europe, medical crowdfunding might be regarded as marginal when compared to the USA. In Europe, as a result of universal health coverage, residents can benefit from adequate, effective and accessible health services and are financially protected. Although the management of health systems varies greatly across Europe, they all provide universal or nearly universal health coverage for their residents [5]. Universal coverage does not imply covering all medical costs for each individual. Typically, not every resident and not all medical procedures are covered. In the healthcare sector, demand for higher quality care is increasing constantly while the healthcare budget is limited. Nowadays, new medications and innovative medical interventions are appearing on the market quicker than ever. Unmet needs for health care emerge as these new medications and innovative medical procedures are typically not financed by national health insurance funds, due either to insufficient information about their efficacy or the required time-consuming legislative changes [6–11]. Long waiting times (and thus the incentive to use private health care providers instead of public ones) and patient co-payment [12] might also motivate individuals to turn to medical crowdfunding.

This study aims at mapping unmet health care needs manifested in medical crowdfunding campaigns in a healthcare system with universal health coverage. In particular, we explore the most common condition, disease or disorder which prompted individuals to turn to crowdfunding in Germany, where universal coverage is provided through statutory and private health insurance. In addition, we reveal the type and size of health-related expenses that individuals aim to finance via crowdfunding. This study is exploratory in nature; it allows a glimpse into the unmet health care needs of residents in a healthcare system with universal health coverage.

### The German healthcare system

In Germany, health insurance is mandatory for all; residents may choose between statutory health insurance and substitutive private health insurance [13]. In Germany, the share of GDP allocated to health spending was 11.7% in 2019 in comparison with an OECD average of 8.8% [14]. Germany spent the equivalent of USD 6646 per person on health in 2019, compared with an OECD average of USD 4224 [14]. In 2019, public sources accounted for 85% of overall health spending, the third highest among the (Organisation for Economic Co-operation and Development) OECD countries [14]. In 2018, Germany was ranked 12th among 35 European countries when measuring the consumer friendliness of the health system by the Euro Health Consumer Index [15].

German statutory health insurance offers comprehensive health care coverage to 90% of the population (73 million people) [16]. Residents earning less than 62.550 euros per year are automatically enrolled in the statutory health insurance system [17]. Only individuals earning more than 62.550 euros per year, self-employed and civil servants can choose which type of health insurance they prefer [18].

In 2020, the statutory health insurance system is administered by 105 non-profit organisations known as *Krankenkasssen* (sickness funds) [19]. These sickness funds are obliged to provide the same minimum level of care and they are not allowed to refuse anyone as a member [20]. In 2020, all sickness funds charge a basic rate of 14.6% of an employee’s gross salary with a monthly ceiling of 4687.50 euros in 2020 [21]. Statutory health insurance covers treatment such as hospital treatment, visits to general practitioners and specialists, rehabilitation (home care and physiotherapy), health checks from the age of 35, cancer screening, medicines, therapies and aids (hearing aids and wheelchairs, dental check-ups, dentures and crowns, orthodontic treatment up to age 18 [18]). In order to avoid overusing the system and to cover some costs of the statutory healthcare system, co-payment charges apply. Most importantly, patients are expected to cover 10% of prescription costs (minimum 5 euros and maximum 10 euros), 10 euros per day for hospital stays (up to a maximum of 28 days per year), 10% of home help costs (minimum 5 euros and maximum 10 euros per day) and 10% of travel costs (minimum 5 euros and maximum 10 euros per journey) [22].

Depending on the provider, individuals may also be charged an additional contribution of up to 1.1%, on average [21]. This additional contribution may entitle individuals to extra treatment not covered by statutory health insurance, such as additional dental care (professional tooth cleaning or dentures), flu and travel vaccinations, cancer screening under 30, osteopathy, homoeopathy, in vitro fertilisation, contraception [18]. Individuals can easily compare the coverage and extra treatments offered by sickness funds by visiting the website of *Krankenkassen Deutschland* or *Tarifcheck* [23, 24].

Moreover, individuals may purchase additional private insurance from health insurance providers to supplement the care they receive under statutory insurance [25]. These supplementary services, depending on the provider, might cover travel health insurance, additional sickness benefits, additional long-term care benefits, better hospital treatment (private hospital rooms, higher fees), additional dental care and alternative medication [18].

### Unmet medical needs

According to the subjective method, unmet medical needs are present if individuals perceive that they have not received the care they needed [26]. According to the objective approach, unmet medical needs are present if it is clinically proven that individuals did not receive the necessary care [27]. In this research, we follow the subjective method and assess both unmet medical needs (e.g., medication, surgery, rehabilitation, treatment-related travel costs) and unmet health-related needs (e.g., difficulties in covering living expenses, given poor health status) self-reported in medical crowdfunding campaigns. In 2012, 3.4% of the EU population reported unmet medical needs according to information extracted from the European Union Statistics of Income and Living Conditions [28].

In the literature, unmet medical needs are explained by two factors: the characteristics of the healthcare system and the attributes of individuals seeking care [29]. The former factor, among others, includes availability of health care services, waiting times before being scheduled for a procedure, referral patterns, and the booking system [29, 30]. Patient co-payments might also create barriers to health care access and thus generate unmet needs, especially given the rising co-payments for pharmaceuticals and outpatient care in several European countries [28, 30]. Fjær et al. [30], using data from the European Social Survey, report that two-thirds of unmet needs for health care can be explained by two factors: waiting lists and appointment availability [30].

The association between unmet medical needs and the characteristics of individuals seeking care is widely researched. In general, studies report that young people, women, individuals with low socio-economic status (e.g., unemployed, homeless, drug users), those with low income and financial constraints, individuals with secondary and tertiary education, and individuals in poor health have a higher likelihood of reporting unmet medical needs [29–40]. Several studies assess unmet medical needs in specific subpopulations, for example, among young adults [41], the unemployed [31], homeless women with children [42], or the elderly [36]. Some other studies map the unmet needs of particular patient groups such as individuals with disabilities [43], patients suffering from cancer [44, 45], people with multiple sclerosis [46] or dementia [47].

Empirical evidence shows that the prevalence of self-reported unmet medical needs varies greatly in Europe [34, 48–50]. Using data from the 2008 European Social Survey, Cylus and Papanicolas [48] show that respondents from Germany report similar levels of perceived barriers to care as respondents from Denmark, France, Poland and Slovenia. Data from the European Union Statistics on Income and Living Conditions (EU*-*SILC) 2009 survey show that the rate of unmet medical needs in Germany is comparable to that of Denmark, Finland, Italy, and Iceland [34]. Another study using data from six different EU-SILC surveys (2008–2013) documents that the percentage of the population reporting foregone medical care in Germany is similar to that of France, Norway, Slovakia and Sweden [49]. The level of unmet needs in Germany is relatively low when compared to the rest of Europe [34, 48, 49]. For Germany, a study among the elderly also finds that the prevalence of self-reported unmet medical needs for health care is low overall [36].

### Crowdfunding for unmet medical needs

Renwick and Mossialos [51] provide a useful *typology* for crowdfunded health projects. They classify health-related crowdfunding campaigns into four types according to the project’s purpose and the funding method. In their typology, crowdfunding projects might finance health expenses, health initiatives, research, or commercial health innovation. Crowdfunding projects in the first category aim at financing a patient’s out-of-pocket expenses for medical services and products, while health initiatives in the second category provide benefit to the wider public or a specific group of people and raise funds, for example, for patient education programmes and disease awareness.

While unmet medical needs are evident when individuals aim at covering their health expenses from donations, all other types of crowdfunding campaigns are related to unmet medical or health-related needs of specific patient populations. Education and awareness-related health initiatives are indications of unmet need for knowledge among patients with a specific disease or disorder, while crowdfunded health projects typically focus on unmet medical needs of patients where treatment is not yet available. Finally, commercial health innovations aim at meeting the drug and therapy (innovative, complementary or alternative) needs of individuals with disposable income.

The *market for crowdfunded health projects* is large and growing exponentially. Given the decentralized nature of the crowdfunding market, estimating the size of the market is challenging. Bassani et al. [1] estimate that by October 2017 health care campaigns raised over $132 million. In contrast, medical crowdfunding campaigns launched on GoFundMe suggest a much larger market size. By 2018, GoFundMe hosted more than 250,000 medical campaigns per year worldwide; these campaigns raised more than $650 million in total [52, 53].

A few studies assess the unmet medical needs of specific patient groups as revealed in crowdfunding campaigns. Studies cover, for example, the unmet needs of patients suffering from cancer [54, 55], patients undergoing orthopaedic surgery [56] or abortion [57, 58], individuals undergoing organ transplants [59, 60] or desiring gender change [61].

Two very recent studies map the health-related needs of a diverse population using crowdfunding; these studies are the closest to the present study. These recent exploratory studies download selected campaigns from GoFundMe for UK and for British Columbia, Canada, respectively [62, 63]. For the UK, the authors analyse 400 campaigns drawn from a non-representative sample (campaigns with larger fundraising target and raising more funds are overrepresented) and point to the barriers in treatment access: limited access to novel therapies in cancer treatment and long waiting times [62]. For Canada, the authors investigate 423 campaigns from British Columbia and show that individuals frequently sought financial support due to gaps in the wider social system: lost wages because of illness and travel-related costs to access care [63]. The authors argue that the commonly perceived limitations of the Canadian health system, such as long waiting times for care and limited access to specialist services did not frequently motivate individuals to seek help from the crowd [63].

Crowdfunded health projects reflect only a small fraction of unmet medical and medical-related needs. In general, *younger adults with higher digital literacy* launch crowdfunding campaigns. Berliner and Kenworthy [3] report that crowdfunding campaigns are typically launched by individuals who have better reading and writing skills, and who have mastered good medical, social media and technical literacy. Snyder et al. [64] also argue that crowdfunding is used by relatively privileged members of society, those being digitally literate and having large social networks. Indeed, large social networks play an important role in reaching the fundraising target; sharing campaigns online through social media sites such as Twitter or Facebook increases the probability of success [56, 65–67]. At the same time, membership of marginalized race and gender groups decreases the probability of reaching the fundraising target; the average donation amount is lower among these marginalized groups [68].

Perhaps the most important critique of crowdfunding is that the less privileged are squeezed out of the crowdfunding market; they not only launch proportionately fewer campaigns, but they also receive less by way of donations per campaign [54, 61, 64, 68]. Fundraising campaigns for medical care reveal and reinforce health and social inequalities [54, 61, 64, 68]. The unmet medical needs of the most needy remain unmet even after launching crowdfunding campaigns. In this way crowdfunding creates an unequal and biased marketplace, thus fuelling health inequities and widens the gap in society [54, 64, 68].

## Methods

### Sample

Donation-based crowdfunding platforms are screened in Germany, the most highly populated country in Europe [69]. On the one hand, the more populated a country is, the higher the chance that individuals search for financing of additional health needs. On the other hand, Germany has a universal healthcare system, the target system of this research. As argued before, the vast majority of residents are enrolled in mandatory state health insurance, which covers a wide array of health care services. Nevertheless, some medical costs are not covered (e.g., patient co-payments, several alternative and complementary therapies, medical interventions with a low expected success rate, experimental therapies) which might motivate individuals to turn to crowdfunding.

In Germany, as of May 2018, three large donation-based crowdfunding platforms offered individuals the opportunity to launch crowdfunding campaigns to cover their medical expenses: Leetchi, Betterplace, and Gynny [70]. On Leetchi, as of 4 May 2018, the time of screening crowdfunding platforms for eligibility, 560 projects were listed in the category of Medicine (*Medizin*) [71]. On Betterplace 629 crowdfunding campaigns were launched in the category of Health (*Gesundheit*) in Europe [72]. As compared to Leetchi, Betterplace maintains a strong focus on campaigns launched by non-profit organizations, such as municipalities, hospitals, and foundations; the number of crowdfunding campaigns launched by individuals was rather exceptional. On Gynny 2372 projects were listed covering a wide array of categories [73]. Although Gynny is listed as a crowdfunding platform on Crowdfunding.de, the platform is designed very differently from typical donation-based crowdfunding platforms. On Gynny individuals can donate through online shopping at partner shops without paying extra charges; they simply need to insert the code of the crowdfunding campaign they wish to support. In this research, crowdfunding campaigns were downloaded from Leetchi; typically, individuals launch campaigns there and its design is similar to many other donation-based crowdfunding platforms.

From the 560 campaigns listed on Leetchi in the category of medicine [71] we excluded those which were unrelated to health. The excluded campaigns were identified through text mining. We built a vocabulary of 505 health-related German words; the vocabulary included words such as diagnose, sick, medicine, medication, doctor, therapy, pain, cancer, treatment, cure, care, and operation and all related compound words. From the 560 crowdfunding campaigns, 164 did not meet this inclusion criterion; the text of these campaigns did not include any of the 505 health-related words defined in the vocabulary.

In addition, from campaigns containing at least one word from the vocabulary, the following were excluded: 1) duplicates; 2) campaigns written in a language other than German; 3) campaigns without any text; 4) campaigns covering non-health related needs of refugees, the homeless or hungry; 5) campaigns involving medical care for animals. Campaigns entitled “Illness of Kunz Walter” or “Medical help” are typical examples of campaigns excluded due to empty campaign descriptions [74, 75]. The campaign entitled “Humanitarian aid for refugees in Europe” is a prototype of a campaign excluded due to non-health related needs [76]. As a result of these additional exclusion criteria, 16 crowdfunding campaigns were excluded. The final sample thus included 380 crowdfunding campaigns.

### Text mining

In this exploratory research, in order to develop categories for which kind of condition, disease or disorder people asked for donations, we screened the titles of the campaigns. During this screening, we developed a vocabulary with keywords (e.g., cancer, mobility, mental disorder) which allowed us to identify the health problem. When developing the vocabulary, we acknowledged that in German it is very common to form compound words—words which assemble several words at the same time to form one word. The number of associated words is unlimited; and sometimes the new word has a completely different meaning. Thus, we first extracted all words which included the keyword, and then we screened the list of the extracted compound words and excluded the irrelevant ones (i.e., changed meaning). We added the relevant compound words to the vocabulary of keywords. Using the text mining package tm in R we identified those campaigns which included any of the words in the extended vocabulary. In order to do so, first we ran some basic text transformation and text cleaning functions and then we built a term-document matrix including all the words in the vocabulary. Finally, we screened the text of the unclassified campaigns and added new health-related keywords to the vocabulary, and repeated the procedure specified above.

In addition to the condition-specific vocabulary, we also developed a vocabulary which allowed us to identify the health-related expenses that individuals aimed at covering from donations. The vocabulary was developed in the same way as specified above, albeit with different key words (e.g., medication, cost of therapy, travel, accommodation, cost of living, holidays).

Finally, by extracting part of a text string based on position in the text string we extracted funding needs as stated in the textual description of the crowdfunding campaigns.

### Manual data extraction

Once the health-related campaigns were identified, we extracted three kinds of information manually from the textual descriptions. First, we read each campaign text carefully and validated the condition, disease or disorder which motivated individuals to seek additional funding. In the case of misspecification, we assigned a new motive for crowdfunding (manual validation). In total, we validated 35 health problems listed at least twice and 18 health problems listed only once.^1^ Second, we extracted the costs type that individuals aimed at covering from donations. The most important cost types identified were as follows: medication, surgery, therapy, medical equipment, treatment-related travel and accommodation, living expenses, holidays, medical research and patient education. Third, we identified whether individuals sought funding for treatment abroad or for non-residents.

## Results

From the 560 crowdfunding campaigns, in total, 180 did not meet one of the inclusion criteria. As a result, the final sample included 380 crowdfunding campaigns.

### The health problem

In several crowdfunding campaigns we identified more than one condition, disease or disorder which prompted individuals to ask for donations. Individuals listed one to six conditions per crowdfunding campaign. In 18 campaigns, although the campaign was evidently health-related and the cost to be covered from the donations could be identified, the condition was not specified. In the majority (62.63%) of campaigns (238 out of 380), individuals listed one specific reason. In 25% of campaigns (95 out of 380), individuals specified two conditions. In 20 campaigns (5.26%) three conditions, in seven campaigns (1.84%) four conditions, and in one campaign five conditions were listed. As a maximum, individuals mentioned six different conditions (*n* = 1).

The most frequent conditions, diseases or disorders which motivated individuals to ask for donations are shown in Table 1; the last column of the table provides information about the cost to be covered. As shown in Table 1, the most frequent health problems include cancer, mental disorder, disability, accident, lipoedema, genetic disorders and rare diseases, elderly and dementia, sclerosis, and oral health.

**Table 1:** The nine most frequent conditions in crowdfunding campaigns

Rank	Health problem	Number (%)	Description of the funding need
1	Cancer	101 (26.58%)	Therapy (*n* = 42); living expenses (*n* = 26); treatment-related travel costs (*n* = 8); support for institutions involved in cancer care (*n* = 7); treatment-related accommodation (*n* = 5); cancer research (*n* = 4); patient education (*n* = 4). In five campaigns funding was requested for therapy abroad, while in 12 campaigns therapy for foreigners was listed.
2	Mental disorder	34 (8.94%)	Animal-assisted therapies (*n* = 11); living expenses (*n* = 11); various therapies not involving animals (*n* = 7); support for institutions involved in mental care (*n* = 2); treatment-related travel costs (*n* = 2), patient education (*n* = 2); holidays to improve mental health (*n* = 2). Treatment for foreigners (*n* = 2) and treatment abroad (*n* = 1) were less frequently requested.
3	Disability	26 (6.84%)	Medical equipment to facilitate mobility (*n* = 15); other medical equipment (e.g. bed for the disabled, therapeutic chair) (*n* = 3); therapy (*n* = 4); living expenses (*n* = 3); treatment-related travel costs (*n* = 2); support for institutions engaged in disabled care (*n* = 2).
4	Accident	23 (6.05%)	Therapy (*n* = 5); handicapped accessible car (*n* = 5); living expenses (*n* = 4); treatment-related travel costs (*n* = 3); treatment-related accommodation (*n* = 3); surgery (*n* = 2); dental treatment (*n* = 2); legal procedures (*n* = 2). In a few cases individuals requested funding for treatment abroad (*n* = 2), or to finance treatment for a non-resident (*n* = 4).
5	Lipoedema	22 (5.79%)	Surgery to remove fat tissues (*n* = 19); plastic surgery (*n* = 1); holidays (*n* = 1); patient education (*n* = 1); legal procedures (*n* = 1).
6	Genetic disorders and rare diseases	20 (5.26%)	Therapy (*n* = 12); living expenses (*n* = 7); medical equipment to facilitate mobility (*n* = 5); medical research (*n* = 4); medication (*n* = 3), treatment-related travel expenses (*n* = 2). Treatment for a foreigner was requested in three cases (*n* = 3), while treatment abroad only in one case (*n* = 1).
7	Elderly and dementia	19 (5.00%)	Support for an existing elderly care institution (*n* = 6); living expenses (*n* = 6); establishing a new elderly care institution (*n* = 3); medication (*n* = 3); dance and movement therapy to prevent dementia (*n* = 2); Alzheimer research (*n* = 1); transportation for the elderly (*n* = 1); studies in elderly care (*n* = 1). In a few cases donations were asked for a foreigner (*n* = 3).
8–9	Sclerosis	15 (3.95%)	Living expenses (*n* = 4); research (*n* = 4); medical equipment to facilitate mobility (*n* = 2); therapy (*n* = 2), medication (*n* = 2); support for an institution where patients with multiple sclerosis are treated (*n* = 1).
8–9	Oral health	15 (3.95%)	Dental treatment (*n* = 13); orthodontal treatment (*n* = 2).

Around one fourth of crowdfunding campaigns (101 out of 380; 26.58%) were related to *cancer/tumour*. Table 2 shows the cancer type by body location or system; this information could be extracted only for around half of the campaigns (51 out of 101); no details were provided in the remaining campaigns. Malignancies of the brain, breast, gastrointestinal tract and leukaemia were the leading cancer indications for crowdfunding. Most commonly individuals asked for donations for various therapies not financed by the health insurance fund (*n* = 42), including alternative therapies, scientifically poorly supported therapies and innovative therapies such as therapies with new substances, micro-immune therapy, Methadon-therapy, and stem cell infusion. Immunotherapy and rehabilitation after surgery were also requested several times. The second most common cost element individuals aimed at covering from the donations were living expenses (*n* = 26). Cancer is a chronic condition [77] which puts a significant financial burden on families due both to patient co-payment (medication, immune strengthener) and lost income.

**Table 2:** Cancer type by body location or system

Cancer type by body location/system	Number of campaigns	% of campaigns (out of 51)
Brain tumour	15	29.41%
Breast cancer	11	21.57%
Leukaemia	10	19.61%
Gastrointestinal/digestive	10	19.61%
Lung cancer	6	11.76%
Gynaecologic (ovarian, cervical, etc.)	5	9.80%
Bone cancer	3	5.88%
Skin cancer	3	5.88%
Prostate cancer	1	1.96%
**Total**	**64**	**> 100%**

Cancer type could be extracted for 51 campaigns only. Some crowdfunding campaigns covered more than one cancer type. As a result, the textual description of the 51 campaigns included 64 cancer types in total

The second most frequent health problem listed in around one-tenth of crowdfunding campaigns was *mental disorder*, typically depression (*n* = 34, 8.94%). Those suffering from mental disorder most frequently sought additional funding for animal-assisted therapies or living expenses. Funding for various therapies such as psychotherapy or infusion therapy was also often requested.

*Disability* was the third most frequent motive for crowdfunding; individuals with a wide array of disabilities and their families were in financial need (*n* = 26, 6.84%). The 26 disability-related campaigns shown in Table 1 can be explained by reasons other than genetic disorder and rare disease (*n* = 20), autism spectrum disorder (*n* = 8), paresis (*n* = 5), cerebral palsy (*n* = 2) and cases where animal-assisted therapy was requested (*n* = 26); these severe disabilities are listed separately and excluded from this category. In this category disability covered, for example, brain damage, severe asthma, severe epilepsy, cancer-related disability, and spinal cord or back injury. In the majority of the campaigns, individuals requested funding to facilitate their mobility (electric wheelchairs, wheelchair-accessible vehicles, handicapped-accessible homes).

*Accident* was ranked as the 4th most frequent cause for medical crowdfunding (*n* = 23, 6.05%); these campaigns were posted to provide relief from the severe consequences of a past accident. From the donations individuals aimed to cover a wide array of expenses, such as handicapped-accessible cars, living expenses and various therapies, for example, physiotherapy, rehabilitation, and Adeli-therapy.

The 5th most frequent medical condition mentioned was *lipoedema* (*n* = 22, 5.79%). Lipoedema is a disorder with symptoms of swelling and enlargement of the lower limbs; an abnormal amount of subcutaneous fat is deposited under the skin [78]. Genetic and hormonal factors contribute to the risk of developing lipoedema [78]. As of now no effective treatment for lipoedema exists; only symptoms can be alleviated. In crowdfunding campaigns individuals almost exclusively requested funding for surgery to remove fat tissues, arguing that the health insurance fund does not cover the cost of the desired intervention.

*Genetic disorders and rare diseases* were mentioned in 20 out of 380 campaigns (5.26%) and ranked in the top six. Down syndrome was listed in three campaigns, and Rett syndrome in two campaigns. Other genetic disorders and rare diseases were mentioned only once. These covered a wide array of conditions, such as Ehlers-Danlos Syndrome, Hodgkin’s Syndrome, Lesch-Nyhan Syndrome, and Li-Fraumeni Syndrome. Those suffering from genetic disorders and rare diseases requested funding for diverse activities. Various therapies, such as physiotherapy, therapy with animals, innovative and scientifically poorly supported therapies were high on the wish list, followed by living expenses, and medical aids to increase mobility.

The 7th most frequent medical condition mentioned in crowdfunding campaigns was *dementia and elderly care* (*n* = 19, 5.00%). Dementia and old age in general are associated with poorer health status and several symptoms; symptoms might be so severe that they interfere with daily life. Crowdfunding campaigns were initiated with diverse purposes, among others to support an existing elderly care institution and to cover living expenses.

Both *sclerosis* and *oral health* urged individuals to launch crowdfunding campaigns in 15 cases (3.95%). Lateral sclerosis (the death of neurons controlling voluntary muscles) and multiple sclerosis *(*damaged insulating covers of nerve cells in the brain and spinal cord) may develop into severe and disabling disease; patients’ muscles become uncoordinated and weak and they might lose their ability to walk [79, 80]. This lifelong condition puts a heavy burden on the patients and their families; individuals most frequently asked for financial support to cover their daily expenses. Funding was also frequently requested for research. Regarding oral health, donations were requested from the crowd for dental or orthodontal treatment not covered by health insurance. In several cases, although health insurance covered some previous treatments, the requested treatment was no longer covered.

Table 3 lists the 10-19th most frequent condition, disease or disorder which prompted individuals to ask for donations from the crowd. The table also provides information about the costs to be covered from donations. Table 4 shows those health problems for which individuals requested funding only in a few campaigns (five or less).

**Table 3:** The 10-19th most frequent conditions in crowdfunding campaigns

Rank	Health problem	Number (%)	Description of the funding need
10–11	Epilepsy	14 (3.69%)	In several cases epilepsy was a comorbid condition in addition to another disease such as autism spectrum disorder (*n* = 3), genetic disorder (*n* = 3) or mental disorder (*n* = 2). In the majority but not all cases epilepsy was a disabling condition (8 out of 14 cases). Patients with epilepsy requested donations for various therapies such as Adeli, swimming or innovative therapy (*n* = 6). Epilepsy watch dogs were high on the wish list (*n* = 8). In some other cases funding was requested to increase mobility with the help of a special needs bike and a stair lift (*n* = 2); to support research in fields where epilepsy is a comorbid condition (*n* = 2); and patient education (*n* = 1). In comparison with other categories, although funding was less frequently requested for living expenses (*n* = 1) and treatment-related travel costs (*n* = 1), donations were more frequently asked to finance treatment abroad (*n* = 6).
10–11	Prosthesis & orthosis	14 (3.69%)	Individuals turned to crowdfunding with prosthesis or orthosis related problems mostly to cover sport prostheses and other very expensive prostheses (*n* = 5), or special therapy for children with orthosis (*n* = 2), none of them being covered by the insurance fund. In some other cases financing was requested to cover treatment-related travel (*n* = 3) and accommodation expenses (*n* = 2), living expenses (*n* = 2), or to install a stair lift facilitating the mobility of an individual with prostheses (*n* = 1). In three cases funding was requested to cover prosthesis-related expenses for patients outside Germany (*n* = 3), including a hospital in Tanzania to produce prostheses.
12	Eye, Blind	13 (3.42%)	In this category donations were requested as a result of various eye problems. From the donations, individuals aimed to cover the living expenses of a family with a blind member (*n* = 4) and the cost of eye surgery not financed by health insurance (*n* = 4). Funding was requested for therapy not covered by the insurance fund in three cases (*n* = 3): electro-acupuncture therapy (*n* = 1), dubious therapies for blind children (*n* = 1), reason unspecified (*n* = 1). One individual wished to go on holiday before becoming completely blind (*n* = 1). Treatment abroad was requested in two cases (*n* = 2), while eye surgery for a foreign child in one case (*n* = 1).
13	Transplants	12 (3.39%)	In this category individuals turned to crowdfunding, for example, to finance surgery (*n* = 3): kidney transplant for a foreigner (*n* = 1) or hair transplant not covered by the insurance fund (*n* = 2). Funding was requested for therapy as frequently as for surgery (*n* = 3): stem cell infusion therapy, micro-immune therapy, and doctor’s visits. The desire to finance living expenses was also mentioned several times (*n* = 3). Covering the cost of transplants for relatives or acquaintances living outside Germany was more frequently mentioned in this category than in the others (*n* = 5; 41.67% of all transplant-related campaigns).
14	Plastic surgery	9 (2.37%)	Funding was requested from the crowd for a variety of aesthetic surgeries; breast augmentation (*n* = 3) and breast reduction (*n* = 2) were on top of the list. The remaining campaigns listed removal of excess skin from the abdomen (*n* = 1), rhinoplasty (*n* = 1) and skin reconstruction (*n* = 1). In one case funding was requested for reconstructive surgery for a relative outside Germany (*n* = 1).
15–17	Weight/Obesity	8 (2.11%)	Overweight individuals requested funding for surgery to remove excess fat (*n* = 1) and/or excess skin (*n* = 4), to install an intragastric balloon (*n* = 1), to buy weight loss products (*n* = 1) or a special needs bicycle for an overweight premature child (*n* = 1).
15–17	Autism spectrum disorder	8 (2.11%)	In the majority of cases, families with children suffering from autism spectrum disorder asked for financial support to ease their everyday lives. In particular, individuals requested funding for animal-assisted therapy (*n* = 4), treatment-related travel expenses (*n* = 2), living expenses (*n* = 2), therapy bicycle (*n* = 1), and legal process to support the mother of an autistic child (*n* = 1). Funding was requested for research in one campaign (*n* = 1). Treatment abroad was mentioned in two campaigns (*n* = 2).
15–17	Heart problems	8 (2.11%)	Patients with heart problems turned to crowdfunding to finance their heart surgery (*n* = 3); their therapy (*n* = 2) and their medication (*n* = 2). Some other motives included a special needs chair (*n* = 1), treatment-related travel costs (*n* = 1), living expenses (*n* = 1), holidays (*n* = 1), and financial support for a heart centre (*n* = 1).
18–19	Diabetes	7 (1.84%)	Individuals with diabetes asked for donations for a wide array of expenses: holidays (*n* = 3); living expenses (*n* = 2); surgery to reduce being overweight and to improve vision (*n* = 2); electric wheelchair (*n* = 1). Funding was requested for a non-resident in one case (*n* = 1).
18–19	Orthopaedics	7 (1.84%)	Individuals with orthopaedic problems requested funding for various expenses: orthopaedic interventions no longer supported by health insurance (*n* = 3), special needs bike with orthopaedic features (*n* = 1), accommodation and travel-related expenses for a series of surgeries financed by insurance (*n* = 1), lawsuits against an orthopaedist (*n* = 1), and opening an orthopaedic clinic in Afghanistan (*n* = 1).

**Table 4:** Less frequently listed conditions in crowdfunding campaigns

Rank	Health problem	Number (%)	Description of the funding need
20–23	In vitro fertility treatments	5 (1.32%)	Couples asked for financial support from the crowd for in vitro fertilization when the costs were not covered by the health insurance fund (low chance of successful fertilization, treatment available abroad only).
20–23	Paresis	5 (1.32%)	Paresis includes both hemiparesis (weakness of one entire side of the body) and tetra-paresis (complete paralysis of the body from the neck down). Individuals with paresis requested donations either for therapy (*n* = 3) or for equipment to facilitate their mobility (*n* = 2).
20–23	Stroke	5 (1.32%)	In addition to therapies (*n* = 2) and equipment to facilitate patients’ mobility (*n* = 2), donations were requested to cover living expenses (*n* = 2).
20–23	Brain damage	5 (1.32%)	Funding was exclusively requested for therapy (*n* = 5), mostly for therapy abroad (3 out of 5 cases).
24–27	Gender change	4 (1.05%)	Changing gender from male to female (*n* = 3), from female to male (*n* = 1).
24–27	Inflammatory lung diseases	4 (1.05%)	Inflammatory lung disease includes, among others, chronic obstructive pulmonary disease and asthma.
24–27	Autoimmune diseases	4 (1.05%)	Autoimmune diseases affecting diverse organs.
24–27	Kidney problems	4 (1.05%)	Kidney transplant (*n* = 1); dialysis (*n* = 1), unspecified (*n* = 2).

The conditions listed in this table are followed by problems with the gastrointestinal system (*n* = 3, 0.79%), alcohol dependence-related problems (*n* = 3, 0.79%), allergy (*n* = 3, 0.79%), problems with the lung system other than inflammatory lung diseases (*n* = 3, 0.79%) and hunger in developing countries (*n* = 3, 0.79%). Conditions mentioned twice include cerebral palsy (*n* = 2, 0.53%), heart attack (*n* = 2, 0.53%), and AIDS (*n* = 2, 0.53%). Conditions mentioned once (*n* = 1, 0.26%) include rare metabolic disease, chronic fatigue syndrome, inflammatory bowel disease, endometritis, hand and finger disease, hip dysplasia, hearing loss, scoliosis (curvature of the spine), lots of body hair, chronic headache, rheumatoid arthritis, limited motoric skills, neurodermatitis, excessive sweating, cervical disc disorder, problems with oesophagus, infected wound, and baby delivery abroad

### Costs to cover

Table 5 shows the 15 most frequent health-related costs for which individuals requested funding on Leetchi. The last column of Table 5 provides some additional information on the cost element. As shown in Table 5, the most frequent medical expense individuals aimed to cover from the donations was related to *therapy*; financial support for therapy was requested in almost one-fourth of the campaigns (*n* = 90; 23.68%). The second most frequent cost type for which individuals asked for donations were *living expenses* (*n* = 77, 20.26%). Living expenses might manifest in various forms, such as paying bills or a rental fee, obtaining a driving licence, house renovation, car costs for going to the doctor or work, removing mould professionally, and leisure activities. Typically, the underlying health problem put such a heavy burden on families, partly due to lost income, partly due to financing additional medications and therapies, that they turned to the crowd to ease their financial burden.

**Table 5:** The fifteen most frequent cost types in crowdfunding campaigns

Rank	Cost type	Number (%)	Comment on the cost type
1	Therapy	90 (23.68%)	Individuals frequently asked for financial support to cover costs of alternative therapy (*n* = 11), rehabilitation (*n* = 9), innovative therapy (*n* = 5), immune therapy including micro-immune therapy (*n* = 5), physiotherapy (n = 4), Adeli-therapy (*n* = 4), dance therapy (n = 3), stem cell infusion therapy (*n* = 2), and other infusion therapies (n = 2).
2	Living expenses	77 (20.26%)	Highly diverse funding request related to everyday life.
3	Support for an institution	39 (10.26%)	In Germany, youth centres and institutions engaged in elderly care and cancer care were the most popular targets of donations. In almost half of the cases, funding was requested for an institution in developing countries (n = 18), typically for establishing health centres, for supporting the volunteer work of various medical professionals, and for easing the life of the most needy (hungry, disabled, seniors, refugees).
4	Equipment to facilitate mobility	37 (9.74%)	Electric wheelchairs (n = 7), handicapped accessible homes (n = 8), handicapped accessible cars (n = 12), prostheses and orthopaedic equipment (*n* = 7), and special needs bike (3) were the most frequently demanded by individuals. Underlying conditions varied greatly, from cancer and genetic disorder to paresis and sport injury.
5	Medication	30 (7.89%)	Medication was frequently requested by patients suffering from cancer (*n* = 13), genetic disorder or rare disease (n = 3) and injuries (*n* = 3).
6	Animal-assisted therapy	26 (6.84%)	Assistance dogs trained to aid a disabled individual were highly demanded (n = 11). Most in demand were guide dogs to assist the blind or visually impaired, but medical alert dogs and psychiatric service dogs were also on the wish list. Dolphin therapy, an intervention involving dolphins, was popular among families with severely disabled children (*n* = 10). Several campaigns aimed to cover the costs of equine-assisted therapy, typically with horses, to improve physical and mental health of children (*n* = 7).
7–8	Treatment-related travel cost	25 (6.58%)	These costs were frequently requested by cancer patients (n = 8), and by individuals experiencing difficulties while travelling due to their prosthesis and orthosis (n = 3), their disability (*n* = 3) or a recent accident (*n* = 3).
7–8	Surgery	25 (6.58%)	In almost half of the cases financial support was requested for a foreigner, for a patient not covered by the German health insurance system (n = 11). Surgery abroad was requested only in three cases (n = 3). Insured individuals requested funding either for innovative surgery or for interventions declined by the health insurance fund (e.g. hair transplant, abdominal sweating). The underlying condition varied greatly, from heart, eye and back surgery to kidney and hair transplants. Donations were also asked for surgery to treat headache, abnormal sweating and pseudarthrosis.
9	Excess fat/skin removal	24 (6.32%)	The underlying condition was either lipoedema (n = 19) or being overweight (n = 5). As research on lipoedema is limited [81]; as of now the only treatment that seems to be effective in reducing the build-up of fatty tissue is a procedure called tumescent liposuction. Until recently these liposuction interventions were excluded from the list of services financed by statutory health insurance and thus from the list of private health insurance companies [82]. Individuals typically turned to crowdfunding either to avoid the bureaucratic procedure of application or after their application was declined.
10	Patient education	18 (4.74%)	In educational crowdfunding campaigns individuals typically asked for financial support to increase knowledge about specific health problems such as mental disorder, lipoedema, female genital mutilation, hand and finger disease, or healthy lifestyle.
11–13	Research	15 (3.95%)	Research projects covered a wide array of health conditions, such as cancer (n = 4), multiple sclerosis (*n* = 4), genetic disorders and rare diseases (n = 4), epilepsy (n = 2), chronic fatigue syndrome (m = 1), and Alzheimers disease (n = 1).
11–13	Dental or orthodontal treatment	15 (3.95%)	In the majority of cases, funding was requested for dental treatment not included in the health insurance plan of individuals. In some cases treatments were related to losing teeth as a result of the side effects of cancer treatment (n = 3) or accident (n = 2).
11–13	Holidays, presents	15 (3.95%)	Donations with the aim of pleasing family members or friends suffering from a disease or disorder. In the majority of cases they aimed to collect money for a surprise holiday, in a few cases for a unique present, such as a T-shirt of the patient’s favourite football club.
14	Treatment-related accommodation	14 (3.68%)	Covering treatment-related accommodation costs typically abroad.
15	Medical aids and devices	13 (3.42%)	Any kind of aid, device or material aimed to be used for medical purposes. Among others, the wish list included special needs chair (n = 3), bed for disabled people (*n* = 1), blood glucose tester (*n* = 1), blood gas analyser (n = 1), defibrillator device (n = 1), chemicals for sterilization (n = 1), and antibody-drug conjugate (n = 1).

*Note:* In the remaining cases, individuals requested donations for plastic surgery (n = 9, 2.37%); healthcare education and training (n = 7, 1.84%); health-related legal procedures (n = 6, 1.58%); in vitro fertilization (n = 5, 1.32%); and gender change (*n* = 4, 1.05%)

In one-tenth of the campaigns, individuals requested financial support for an institution (*n* = 39, 10.26%). Almost as popular were requests for donations to facilitate patients’ mobility (*n* = 37; 9.74%). Families also often asked for donations for medication (*n* = 30, 7.89%), arguing that drug costs put heavy burden on their budget in addition to the burden of the disease, disorder or condition.

### Treatment for foreigners and treatment abroad

Regarding *geographic coverage*, the huge majority of crowdfunding campaigns did not list any country, city or nationality in the campaign description (*n* = 304, 80%). These projects were typically posted by residents to fund health care services delivered in their neighbourhood in Germany. In total, almost 13% of crowdfunding projects (*n* = 49, 12.89%) involved a *foreign country* for reasons other than holidays; funding was requested either for patients residing abroad and thus not covered by the German health insurance fund (*n* = 31), or for a health initiative in a developing country (*n* = 18). Developing countries were involved in 35 out of the 49 projects; countries within the European Economic Area (EU, Norway and Switzerland) were mentioned in five crowdfunding projects; other European countries, e.g. Bosnia and Herzegovina, Russia, and Turkey were mentioned in eight crowdfunding campaigns. High-income countries outside Europe were mentioned in only one crowdfunding campaign; an individual sought funding for medical intervention offered in the USA only. Although the underlying conditions varied greatly, for non-resident patients the three most frequent conditions included cancer (*n* = 12), transplants (*n* = 5) and accidents (*n* = 4).

Donations were asked for treatment abroad in 27 out of 380 cases (7.11%). Typically, individuals asked for donations to finance therapy not available in Germany (*n* = 12), such as Adeli-therapy offered in Slovakia, new innovative therapies only offered in the US, or stem cell infusion therapy. Animal-assisted therapies involving dolphins were high on the wish list (*n* = 10). Surgery outside Germany was requested only in three cases. Although the underlying conditions varied greatly, three conditions were frequently mentioned: disability (*n* = 10) with a comorbidity of epilepsy in half of the cases, cancer (*n* = 5) and brain damage (*n* = 3). Other disorders, diseases or conditions included cerebral palsy, paresis, genetic disorder, autism, prosthesis, orthopaedic intervention, accident, and mental disorder.

### Funding need

**Fig. 1:**
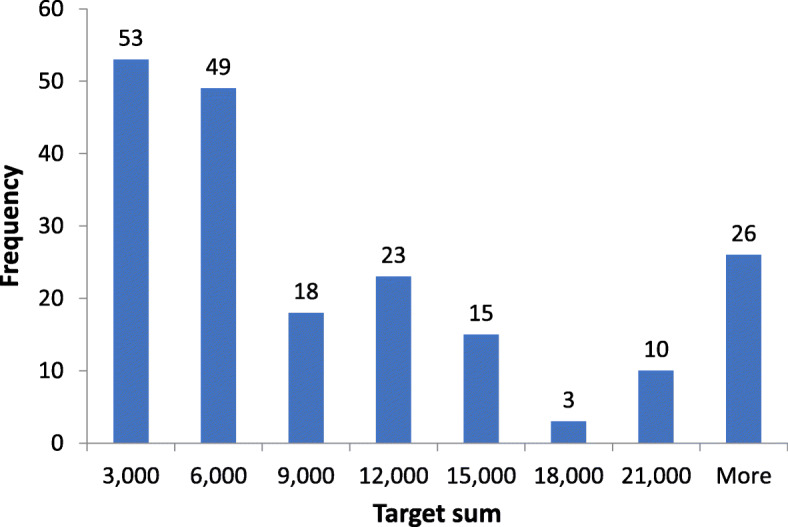
Histogram of funding need

Funding need was stated only in 197 out of 380 crowdfunding campaigns (51.84%). In the remaining cases (*n* = 183, 48.16%) campaign holders typically wrote that donors could give as much as they want. Table 6 shows the descriptive statistics of funding needs, while Fig. 1 plots the histogram of funding needs for campaigns with a target sum. The mean funding need was €14,166 after excluding two outliers with a target sum of €1 and €6 million. (The former campaign aimed to ease the life of patients with hyperhidrosis and bromhidrosis through surgery, innovative medical intervention and financial support, while the latter asked for donations for a researcher without any publications on Google Scholar.) Campaigns with lower funding needs were more popular; the funding need was €6000 or lower in more than half of cases (102 out of 198). Nevertheless, there were a few campaigns with large funding needs: 18 campaigns aimed at collecting more than €30,000.

**Table 6:** Descriptive statistics of funding need

	Crowdfunding campaigns with a target sum (*n* = 197)	Crowdfunding campaigns with a target sum, excluding 2 outliers (*n* = 196)
Average	49,555	14,166
Median	6000	6000
Min	70	70
Max	6000,000	250,000
IQR	3000 – 14,402	3000 – 14,450
St dev	432,850	29,340

Funding needs were the highest, on average, in the category of elderly and dementia (€40,208, *n* = 12), followed by transplants (€35,840, *n* = 5), cancer (€18,859, *n* = 40), sclerosis (€16,350, *n* = 6), and lipoedema (€15,317, *n* = 15). For the 14 most frequent conditions the full list is shown in Table 7, ordered by average funding need in decreasing order.

**Table 7:** Average funding need (ranked in decreasing order)

Rank by frequency	Health problem	Average funding need	Number of campaigns with a target sum
7	Elderly and dementia	40,208	12
13	Transplants	35,840	5
1	Cancer	18,859	40
8–9	Sclerosis	16,250	6
5	Lipoedema	15,317	15
10–11	Epilepsy	14,038	8
6	Genetic disorders and rare diseases	12,254	7
10–11	Prosthesis & orthosis	11,213	8
3	Disability	10,559	22
8–9	Oral health	10,120	10
2	Mental disorder	7815	19
4	Accident	7075	8
12	Eye, Blind	5743	7
14	Plastic surgery	5533	6

## Discussion

### Unmet needs due to financial strains

On Leetchi, all four *types of crowdfunded health projects* classified by Renwick and Mossialos [51] were present. The huge majority of campaigns aimed at financing expenses for medical services. Not-for-profit health initiatives served as a motive for crowdfunding in around 15% of campaigns in the form of fundraising for medical institutions or charitable organizations, and patient education and disease awareness campaigns. Donations were requested for research less frequently. Commercial health innovation was listed in only one fraudulent campaign [83] discussed in subsection 4.4 in more detail.

Although the expenses that individuals aimed to finance via crowdfunding varied greatly, *unmet medical needs* due to financial strains were listed in almost 80% of crowdfunding campaigns (Table 5).^2^ Across all conditions, donations were most frequently requested for therapies, typically additional or complementary therapy not financed by the health insurance fund. Animal-assisted therapies were also high on the wish list; although these therapies are not always covered by statutory health insurance, they are expected to ease the emotional and physical burden of affected individuals and families. A similar argument can be made for equipment to facilitate mobility.

The medical condition that individuals were suffering from resulted in significant *unmet non-medical needs* as well. Individuals could not pay the bill they received, and they could not go on holidays they desired; their (or their children’s) poor health status typically did not allow them to earn sufficient income. Living expenses was the second most frequently listed cost type in accordance with the financial strain that several disorders and diseases exert on families. This finding is in line with the empirical evidence showing that poor health status may be associated with poor socioeconomic conditions, labelled as social drift or selection in the literature [84]. If affected families enjoyed better socioeconomic status, they could finance these expenses without any difficulty.

### Crowdfunding motives, causes of death, and disease burden

There is a weak association between *the most frequent causes of death* and the condition which motivated individuals to ask for donations. According to the WHO, in upper-middle income countries the top ten causes of death are as follows: ischaemic heart disease; stroke; chronic obstructive pulmonary disease, trachea, bronchus and lung cancer; Alzheimer’s disease and other dementias; lower respiratory infections; diabetes mellitus; road injury; liver cancer, stomach cancer [85]. From the most frequent causes of death only three overlap with the most frequently mentioned motives in medical crowdfunding campaigns: cancer (the most frequent motive for crowdfunding); accident (the 4th most frequent motive for crowdfunding), and elderly and dementia (the 7th most frequent motive for crowdfunding). Regarding cancer, when cancer is screened by type, lung cancer and cancer affecting the gastrointestinal or digestive system amount to more than 30% of cases (Table 2). As a result, these two cancer types alone assure that cancer is among the top ten motives for medical crowdfunding.

There is also no strong association between *the disease burden* measured by disability-adjusted life years and the condition which motivated individuals to ask for donations. In the remaining part of this subsection that association is discussed. First, we show signs of a strong positive association and then we elaborate on those conditions where no association can be found.

*Cancer* is the most frequent motive for crowdfunding, it is named in more than one-fourth of campaigns. According to the Global Burden of Disease (GBD) Study 2017 cancer is the second leading cause of disability worldwide and it exerts enormous emotional, physical and financial strain on patients, families and health systems [86]. Given the high severity of health loss and the related financial burden, it is no surprise that even insured cancer sufferers ask for donations, most frequently for alternative and highly innovative therapies not financed by the health insurance fund. At the same time, in 12 campaigns donations were also asked for uninsured non-residents reflecting the fact that health systems in low- and middle-income countries typically lack resources to manage cancer. In crowdfunding campaigns, the most frequently listed cancer type by body location or system only partly overlaps with the most common types of cancer as listed by the WHO [87]. For example, while lung, breast, and stomach cancer were high on the list in both cases, brain tumour and leukaemia were frequently mentioned in crowdfunding campaigns (Table 2), but not listed as the most common types of cancer by the WHO [87].

The second most frequent medical condition appearing in around one-tenth of crowdfunding campaigns was *mental disorder*, typically depression. WHO lists depression as the leading cause of disability worldwide and as a major contributor to the overall global burden of disease [88]. In Europe, mental disorder is ranked fifth when measuring the overall disease burden with the number of years lost due to ill-health, disability or early death [89]. In a health system with universal coverage those suffering from mental disorder asked for donations most frequently for animal-assisted therapies to improve their mental health and for living expenses to compensate for lost income.

In Europe, *musculoskeletal disorders* are ranked as the third most disabling condition when measured by disability-adjusted life years [89]. Musculoskeletal disorders affect human body movement or musculoskeletal system; these are injuries and disorders characterised by limited mobility, dexterity, and functional ability [90]. Musculoskeletal condition-related disabilities and accidents resulting in injuries were among the most frequent conditions prompting individuals to turn to crowdfunding in Germany, reflecting how burdensome and severely disabling these conditions are. Donations were the most frequently requested for easing mobility, for example, wheelchair-accessible vehicles and handicapped-accessible homes. Similarly, therapeutic fees were also repeatedly mentioned for both musculoskeletal condition-related disabilities and accidents: typically, additional or innovative therapies not financed by the health insurance fund.

In Europe, *neurological disorders* are ranked as the fourth most disabling condition when measured by disability-adjusted life years [89]. Among others, these disorders include epilepsy, Alzheimer’s disease and other dementias, Parkinson’s disease, multiple sclerosis, and cerebrovascular diseases. Elderly and dementia, sclerosis, and epilepsy were among the most frequent conditions prompting medical crowdfunding. In addition to living expenses, donations were asked for elderly care institutions, for research about sclerosis, and for watch dogs and additional and innovative therapies for patients with epilepsy.

At the same time, neither cardiovascular diseases, having the highest overall disease burden, nor several other diseases characterized by the highest number of years lost due to ill-health, disability or early death (chronic respiratory diseases, diabetes, kidney and digestive diseases, substance use disorders, skin and subcutaneous diseases, sense organ diseases) featured among the most frequent motives for crowdfunding [89]. One possible explanation for this mismatch could be that for these diseases efficient treatments are available for insured individuals, despite the high disease burden.

Moreover, oral health, lipoedema, genetic disorders and rare diseases, not listed among leading causes of disability worldwide [89], prompted individuals to turn to crowdfunding relatively frequently. For oral health, individuals typically requested donations from the crowd when treatments were available but not covered by the health insurance fund. For lipoedema, no effective treatment exists: only the symptoms can be alleviated by removing fat (liposuction). In the past, the statutory health insurance typically did not cover the cost of these liposuction procedures, which prompted desperate patients to ask for donations. Very recently, however, the highest decision-making body of the joint self-government of health insurance funds in Germany, the *Gemeinsame Bundesausschus* defined a patient group eligible for liposuction at the expense of statutory health insurance [82]. Children suffering from genetic disorders and rare diseases exert enormous emotional and financial strain on families; families typically live only on one income and ask for donations for therapies not covered by the health insurance fund, either due to their additional or innovative nature. Donations for research activities were also relatively frequently mentioned with the hope that the disorder might soon be cured.

### Statutory financing versus crowdfunding

Medical crowdfunding might be a viable option for those *scientifically proven treatments* which are not financed by the German statutory health insurance system for some reason. When funding is requested for a non-resident, a safety gap exists in a system other than the German statutory healthcare system. When funding is requested for an insured individual, then there is a safety gap: the desired medical intervention is not covered at all or it is not covered by the insurance scheme without additional contributions. Frequently requested scientifically proven treatments include, among others, rehabilitation after injury or cancer, surgery, dental or orthodontal treatments, animal-assisted therapies (excluding dolphin therapy), and therapy with medication. Scientifically proven treatments requested only a couple of times include, for example, microimmuno-therapy for cancer patients (*n* = 2) [91], stem cell transplant for a patient with autoimmune disease [92, 93], antibody-drug conjugate (Adcetris with the active substance of brentuximab vendotin) medication again for a cancer patient [94, 95], and MiraDry therapy for treating excessive underarm sweating [96, 97]. Medical equipment to facilitate mobility might be also listed in this category; although their efficacy is proven they are not financed for some reason.

Policy makers responsible for setting the coverage of the statutory health insurance scheme might consider addressing some of the above-listed safety gaps among insured individuals. In a systematic analysis, the prevalence of medical conditions might be compared with their popularity in medical crowdfunding campaigns. Such a comparison might reveal striking mismatches in the rankings which should act as a warning signal for potential safety gaps. Policy makers should analyse those cases in detail where the condition or desired treatment is mentioned in crowdfunding platforms more often than would be justified on the basis of prevalence rates. One prominent example for such a mismatch is related to lipoedema. As argued above, this mismatch has recently been recognized by the decision-making body of the joint self-government of health insurance funds in Germany, and liposuction is now covered by statutory health insurance for more patients [82]. Given the limited healthcare budget, several safety gaps are acknowledged by the statutory health insurance fund and purposely left with the individual to cover. Prominent examples of such cases include therapies with a low expected success rate (e.g., in-vitro fertilization for women with severe endometriosis) or much higher costs (e.g., orthodontic treatment beyond age 18). Both situations regularly prompted individuals to turn to crowdfunding; as long as the state budget does not allow coverage of such expenses crowdfunding might be a viable option for health financing in these cases.

Medical crowdfunding should be considered as a viable option for *experimental therapies;* without sufficient evidence, statutory health insurance does not cover such therapies. Donations for experimental therapies were requested in four crowdfunding campaigns. Two patients suffering from cancer aimed to finance innovative cancer treatments: chemotherapy combined with a therapy using exosomes (pumping them in the spinal metastasis) [8], and chemotherapy where methadone, an analgesic against cancer pain, is used as a chemosensitizer [9, 98]. Two patients, one suffering from a rare form of Leukaemia [10], the other suffering from a rare genetic disorder (Limb-girdle muscular dystrophy) were collecting donations for taking part in a research programme [11]. The first patient wanted to visit the Children’s Hospital in Seattle and take part in a study for a Car-T-Cell therapy, while the second aimed at participating in a research programmes on cell and gene therapy development for muscular dystrophy at the Experimental and Clinical Research Centre in Berlin.

Medical crowdfunding might also be considered as a viable option for *poorly supported therapies.* Treatments funded from donations might increase the evidence base and thus provide sufficient scientific evidence for their efficacy, which in turn might enable the statutory health insurance scheme to add the therapy to the list and cover the cost in the future. Prominent examples of such therapies include Adeli-therapy, Doman-therapy and NeuroScan Balance therapy. Adeli-therapy was requested in four crowdfunding campaigns; the published evidence on this therapy is scant and involves a small number of participants [99–101]. Doman-therapy was requested in one campaign [102]. This is a therapy offered for children with special needs in Philadelphia [103]. An early study concluded that data so far available are insufficient to justify the system of treatment [104]; no similar study has yet been published afterwards. Similarly, although NeuroScan Balance is offered in several clinics in Germany [105] and was requested by one individual [106], there is no published evidence on Google Scholar.

*Alternative and complementary therapies* can also be considered good candidates for medical crowdfunding. Such therapies include, among others, acupuncture, homeopathy, naturopathy, Chinese and oriental medicine, body movement therapy, music and dance therapy [107]. Alternative and complementary therapies might give comfort and increase the well-being of patients and their families. In the sample, donations were relatively frequently requested for traditional alternative therapies *(n = 11),* while less frequently for dance therapy, typically against Alzheimer’s and other dementias *(n = 3).* Although such therapies are justified from the point of view that it allows patients to feel better and cope better with their medical condition, the budget of the statutory health insurance fund is limited, and thus does not allow it to cover all costs, including the costs of several alternative and complementary therapies. With an unlimited budget, such therapies should be financed. Crowdfunding is a means to attract additional funding to the budget available for healthcare and thus might work as a complementary tool for health care financing.

Medical crowdfunding might also be considered as a viable option for those medical interventions which *increase the well-being of individuals* but cannot be considered as traditional, alternative, or complementary therapies. Such interventions include, for example, plastic surgery improving the appearance of a body part, gender change, and dolphin therapy. Regarding the latter, empirical evidence shows that dolphin-assisted therapies most probably only improve the mood of a child and its family while on vacation [108]. Therapies increasing the well-being of individuals were frequently listed as motives in crowdfunding campaigns: cosmetic surgery was mentioned in nine, gender change in four, while dolphin therapy in ten campaigns. Financing such therapies is evidently beyond the scope of universal coverage. Should such therapies be financed by donations from the crowd? We may let the crowd decide on this one.

*Scientifically unsupported and dangerous* treatments should be neither supported by the statutory health insurance fund nor allowed to be launched on crowdfunding websites. Crowdfunding activity for five such treatments is investigated by Vox et al. [109]: homeopathy or naturopathy for cancer, hyperbaric oxygen therapy for brain injury, stem-cell therapy for brain injury and spinal cord injury, and long-term antibiotic therapy for chronic Lyme disease. Some of these treatments are ineffective (homeopathy for cancer and hyperbaric oxygen therapy for brain injury), while others may result in serious adverse effects (stem-cell therapy for central nervous system injury and long-term antibiotic therapy for chronic Lyme disease) [109–111]. Individuals launched four medical crowdfunding campaigns for such treatments on Leetchi, in particular, for naturopathy to cure cancer. As medical crowdfunding platforms provide a forum for spreading inaccurate information about treatment [110], it would be important to develop patient education initiatives and health policies targeted at potential users of scientifically unsupported and dangerous treatments [111]. Otherwise, false hopes will be raised, there will be longer delays in appropriate care, and the survival rate will decrease.

### Deficiencies in the health care system

In one-fourth of the crowdfunding campaigns (*n* = 96, 25.26%) individuals blamed the sickness fund and referred to gaps in service provision. Those gaps were almost exclusively related to the range of services provided by the sickness funds. Prominent examples of such gaps include additional, alternative and complementary therapies, rehabilitation after injury or cancer, and equipment to facilitate mobility. Accessing alternative, complementary, and novel therapies is highly important for patients with poor life prognosis and those with strong disabilities. In a low number of cases individuals expressed their desire to get access to specific treatment methods which was rejected by the sickness fund. Prominent examples of such rejection include dental and orthodontal care, liposuction intervention and in-vitro fertilization with low expected success rate.

Not all medical needs described in the campaign texts should be considered as a health system gap. Scientifically unsupported and dangerous treatments, and medical interventions increasing the well-being of individuals but not being considered as traditional, alternative and complementary therapies (e.g., cosmetic surgeries, gender change) fall well outside the scope of the statutory health insurance scheme.

A small number of residents sought funds for treatment abroad (*n* = 27, 7.11%). These campaigns were all related to accessing care not available in Germany. Special rehabilitation therapies not available in Germany (*n* = 12, 3.16%) and dolphin-therapies most probably only improving the mood of the child and the family being on vacation (*n* = 10, 2.63%) are prominent examples for such care [108]. Surgeries outside Germany were requested only in three cases (0.79%). No individuals believed that they would have access to better care elsewhere; nobody desired to be treated abroad when the treatment was available in Germany.

In the textual description of the campaigns, individuals complained about long waiting times only in a few cases (*n* = 5, 1.32%). In spite of the complaints and the desire to shorten the waiting times, individuals did not consider the possibility of bypassing the waiting list, for example, by undergoing the medical procedure in a private clinic. Only one fundraiser expressed fears that her health condition would deteriorate further by the time the statutory health insurance takes care of her. Deteriorating health condition was rather mentioned as an argument to raise funds for an alternative treatment which might help improving the health status.

### Fraud and ethical considerations

Out of the 380 campaigns, two were evidently fraudulent campaigns. One researcher, claimed to be the discoverer of mitochondrial intelligence, asked for €6 million in donations to save the lives of millions [83]. This target sum is by far the highest on Leetchi in the category of health; it is twice as high as the target sum of all other medical crowdfunding campaigns in aggregate. In his project description Dorian Treitz claims to be the discoverer of mitochondrial intelligence, a discovery not yet published, which has dramatic consequences for all of us [83]. Nevertheless, the campaign holder had no publications on Google Scholar.

In another campaign, donations were asked for special therapy weeks for blind children for letting them experience seeing without eyes [112]. The organizer, Axel Kimmel, claims that with their innovative technique blind people can visually recognize the world again. According to current knowledge in medicine, although it is possible to sense light without sight as humans may have light-detecting molecules outside of the eyes [113], humans cannot see without eyes; they cannot detect the wavelengths of the electromagnetic spectrum and turn it into visual images. It is also worth noting that this crowdfunding campaign has been described as a magic trick by former trainer Reinhard Hofstetter pointing out that it is all about motivation, beliefs and positive attitudes towards life [114].

In general, the functioning of crowdfunding platforms is conditional upon trust in their legitimacy; this trust is a precondition for donors to participate. As a result, to enhance platform legitimacy, crowdfunding platforms should take a larger role in detecting and punishing fraudulent behaviour. Those campaigns where fraud is evident should not be published on crowdfunding platforms. For published campaigns, easy-to-use tools should be available for individuals to report suspected fraud; such campaigns should then be investigated and removed if indeed deemed fraudulent.

In addition to misusing funds, crowdfunding raises several important ethical concerns. Among others, medical crowdfunding may undermine privacy; to establish credibility, individuals launching a medical crowdfunding campaign must disclose personal health information [65, 115, 116]. Although campaign holders are typically aware of losing their privacy, they are not overwhelmingly concerned; the financial need outweighed the discomfort of publishing personal health information online [116]. In health systems with universal coverage, crowdfunding might introduce market norms that could commodify health care [117]. In countries without universal health coverage, medical crowdfunding might widen health inequities as it benefits relatively wealthy members of society, those being digitally literate and having large social networks [64]. These people rely on medical crowdfunding which will undermine systemic health reforms by delaying or impeding those reforms through alleviating a need that is or should fall on the system to meet [54, 61, 64, 68]. Another ethical concern is using medical crowdfunding for scientifically unsupported or potentially dangerous treatments [109, 118]. Vox et al. [109] report that over 1000 medical crowdfunding campaigns raised money for five different treatments that are unsupported by evidence or are potentially unsafe—more than $6.7 million in total.

### Limitations

This exploratory study suffers from several *limitations* when mapping unmet health care need. Most importantly, many affected individuals may not consider crowdfunding as a viable option for financing their medical expenses and do not launch any crowdfunding campaigns. Thus, unmet health care needs are only mapped for a subpopulation of patients; namely, for those who have mastered better medical, social media and technical literacies and have better reading and writing skills [3, 64]. As a result, children and young adults are overrepresented, while the middle-aged and elderly are underrepresented in the sample when compared to the age distribution of the population in Germany [69], given the higher digital literacy of young adults (as parents of children). Medical conditions and expenses identified in this study should thus be considered as a non-representative snapshot of unmet health care needs.

As a second limitation, the funding need estimates of this study are only indicative of unmet health care and health-related needs. Although unmet needs are evidently present, estimating related funding needs presents a challenge. Affected individuals may not consider crowdfunding as a viable option for financing their medical expenses, and half of those launching a crowdfunding campaign do not state any target sum, merely that donors should give as much as they want.

Third, we mapped unmet health care needs based on crowdfunding campaigns from one platform from one country. Had we extracted information from other crowdfunding platforms and from other countries, we might have identified partly different medical conditions and expenses due to variations in platform settings and expenses universally covered. Nevertheless, the typology proposed in this study should be valid to some extent for all countries with universal health coverage. Finally, we could only filter out evident misuse of funds raised via crowdfunding. We could not control for other forms of fraud such as lying about medical conditions, creating fake campaigns for sick acquaintances, and using donations for purposes other than those indicated in the campaign description [65].

## Conclusions

In this exploratory study we mapped the unmet medical and health-related needs of residents in Germany, in a healthcare system with universal health coverage., We identified the most common conditions, diseases and disorders which prompted individuals to turn to crowdfunding. The nine most common conditions covered almost two-thirds of campaigns. We found that some of these conditions overlap with the most disabling conditions when measured by disability-adjusted life years. Cancer, mental, musculoskeletal and neurological disorders were frequently listed conditions in crowdfunding campaigns while being leading causes of disability worldwide. Nevertheless, there is no strong association between the disease burden and the condition which prompted individuals to ask for donations. Although oral health, lipoedema, and genetic disorders and rare diseases were not listed among the leading causes of disability worldwide, these conditions frequently prompted individuals to turn to crowdfunding.

In Germany, where statutory health insurance provides wide coverage, medical crowdfunding might be considered as a viable option for financing experimental and poorly supported therapies lacking an evidence base, alternative and complementary therapies giving comfort and increasing the well-being of patients and their families, therapies with a low expected success rate (e.g., in-vitro fertilization for women with severe endometriosis) or much higher costs (e.g., orthodontic treatment beyond age 18), and interventions which increase the well-being of individuals but cannot be considered as traditional, alternative and complementary therapies (e.g., cosmetic surgery, gender change, dolphin therapy). Scientifically unsupported and dangerous treatments, such as homeopathy for cancer, should be neither supported by the statutory health insurance fund nor allowed to be launched on crowdfunding websites.

The medical condition that individuals were suffering from resulted in significant unmet non-medical needs as well. This exploratory study revealed that in more than one-fifth of crowdfunding campaigns, individuals sought financial support to cover their daily expenses. Due to their or a family member’s poor health status individuals could not earn sufficient income and thus turned to crowdfunding to address the financial burden caused by poor health beyond medical needs. This finding is in line with the social drift hypothesis − disabled and bad health status is associated with poor socioeconomic status. These campaigns thus were motivated by gaps in the wider social system. Asking donations for daily expenses show that unmet non-medical needs should also be part of the discussion on the burden of ill health and gaps in the social security system.

This study provided a first glimpse into using the textual descriptions of medical crowdfunding campaigns as a supplementary source of information for the statutory health insurance scheme. It offered an innovative insight into the unmet medical and social needs of a non-representative patient population. Although it is too early to formulate relevant policy recommendations based on this exploratory study, further analysis of the most common diseases and disorders listed in crowdfunding campaigns might provide guidance to national health insurance funds in extending their list of funded medical interventions in countries with universal health coverage. Individuals in desperate need launching crowdfunding campaigns with those diseases signal high unmet needs for available but as yet unfinanced treatment. One prominent example of such treatment is liposuction for patients suffering from lipoedema; these treatments were frequently listed in crowdfunding campaigns and might soon be available for patients at the expense of the statutory health insurance in Germany.

Given the exploratory nature of this study, there is a clear need for additional research. Future studies should address the implications of medical crowdfunding for the health status of individuals; the possibility to access a larger pool of alternative, complementary and experimental therapies; the non-desired consequence of assessing scientifically unproven and dangerous treatments; the implications for equity; and the potential gaps in the health care and social security system. In addition, fraud in medical crowdfunding should be kept to a minimum, there is a need for policy recommendations to avoid such fraud.

## Data Availability

All the data analysed during the current study can be downloaded from leetichi.com (https://www.leetchi.com/en/money-pots/medical). For this study, the data was downloaded on 4 May 2018. The dataset and the vocabulary of 505 health-related German words used in this study are available from the corresponding author on reasonable request.

## Abbreviations

AIDS: Acquired Immunodeficiency Syndrome
GBD Study: Global Burden of Disease Study
EU: European Union
EU-SILC: European Union Statistics on Income and Living Conditions
NHS: National Health System
OECD: Organisation for Economic Co-operation and Development
US: United States
WHO: World Health Organization

## Footnotes

1 The health problems listed at least twice were as follows: Accident, AIDS, alcohol dependence, allergy, autism spectrum disorder, autoimmune diseases, brain damage, cancer, cerebral palsy, diabetes, disability, elderly and dementia, epilepsy, eye problems/blind, gastrointestinal problems, gender change, genetic disorders and rare diseases, heart attack, heart problem, hunger, in vitro fertility treatments, inflammatory lung diseases, kidney problems, lipoedema, lung problems other than inflammatory lung diseases, mental disorder, oral health, orthopaedics, paresis, plastic surgery, prosthesis & orthosis, sclerosis, stroke, transplants, weight/obesity.

2 An unmet need was considered as medical if funding was requested for direct or indirect medical expenses: therapy, including animal assisted therapy; equipment to facilitate mobility; medication; surgery, including excess fat/skin removal; dental or orthodontal treatment; medical aids and devices; treatment-related travel and accommodation costs.
